# Letter to the Editor: Response to Luke Fletcher and Philip Peyton regarding “Predictive validity of a novel non-invasive estimation of effective shunt fraction in critically ill patients”

**DOI:** 10.1186/s40635-020-00342-y

**Published:** 2020-10-01

**Authors:** Emma M. Chang, Andrew Bretherick, Gordon B. Drummond, J. Kenneth Baillie

**Affiliations:** 1 Anaesthesia, Critical Care and Pain Medicine, Royal Infirmary of Edinburgh, Edinburgh, EH16 4SA UK; 2 MRC Institute of Genetics and Molecular Medicine, The University of Edinburgh, Edinburgh, EH4 2XU UK; 3 The Roslin Institute and Royal (Dick) School of Veterinary Studies, University of Edinburgh, Easter Bush, Edinburgh, EH25 9RG UK

To the Editor,

We thank Peyton and Fletcher for their interest in our paper, where we devised a method to infer shunt fraction from a single arterial blood gas sample, with the intention to allow clinical research and prediction without the need for additional invasive measurements, as stated both in the abstract and the main text of our report. Peyton and Fletcher have usefully emphasised this point by citing their own work, in which more invasive samples could be taken. However, it is very likely that the correct derivation of the relationship between the relevant components of the equation has been known and used well before this work: see, for example, the diagrammatic explanation shown in our citation 2 [1]. We do not claim that this simple algebraic rearrangement is original.

As we stated in our paper, our conclusion is that the estimated shunt fraction, although imperfect, is an improvement upon P/F. We recommend its use instead of other measures in situations where FIO2 is changing.

We thank the authors for spotting the typo in one of the equations in our paper on Effective Shunt (ES). A set of parentheses were dropped in the published version. We have now corrected this.

Best wishes,

Emma Chang, Andrew Bretherick, Gordon Drummond and Kenneth Baillie

## Data Availability

Not applicable

## References

[1] Drummond GB, Zhong NS (1983). Inspired oxygen and oxygen transfer during artificial ventilation for respiratory failure. British journal of anaesthesia..

